# Three-Dimensional Printed PLA and PLA/PHA Dumbbell-Shaped Specimens: Material Defects and Their Impact on Degradation Behavior

**DOI:** 10.3390/ma13082005

**Published:** 2020-04-24

**Authors:** Joanna Rydz, Jakub Włodarczyk, Jennifer Gonzalez Ausejo, Marta Musioł, Wanda Sikorska, Michał Sobota, Anna Hercog, Khadar Duale, Henryk Janeczek

**Affiliations:** 1 Centre of Polymer and Carbon Materials, Polish Academy of Sciences, 34, M. Curie-Skłodowska St., 41-800 Zabrze, Poland; jwlodarczyk@cmpw-pan.edu.pl (J.W.); mmusiol@cmpw-pan.edu.pl (M.M.); wsikorska@cmpw-pan.edu.pl (W.S.); m.sobota@cmpw-pan.edu.pl (M.S.); ahercog@cmpw-pan.edu.pl (A.H.); khadar.duale@gmail.com (K.D.); hjaneczek@cmpw-pan.edu.pl (H.J.); 2 Polymers and Advanced Materials Group (PIMA), Universitat Jaume I, Avda. Vicent Sos Baynat s/n, 12071 Castellon, Spain; ausejo@esid.uji.es

**Keywords:** (bio)degradable polyesters, hydrolytic degradation, three-dimensional printing, defect, shrinkage after post-processing heat treatment

## Abstract

The use of (bio)degradable polymers, especially in medical applications, requires a proper understanding of their properties and behavior in various environments. Structural elements made of such polymers may be exposed to changing environmental conditions, which may cause defects. That is why it is so important to determine the effect of processing conditions on polymer properties and also their subsequent behavior during degradation. This paper presents original research on a specimen’s damage during 70 days of hydrolytic degradation. During a standard hydrolytic degradation study of polylactide and polylactide/polyhydroxyalkanoate dumbbell-shaped specimens obtained by 3D printing with two different processing build directions, exhibited unexpected shrinkage phenomena in the last degradation series, representing approximately 50% of the length of the specimens irrespective of the printing direction. Therefore, the continuation of previous ex-ante research of advanced polymer materials is presented to identify any possible defects before they arise and to minimize the potential failures of novel polymer products during their use and also during degradation. Studies on the impact of a specific processing method, i.e., processing parameters and conditions, on the properties expressed in molar mass and thermal properties changes of specimens obtained by three-dimensional printing from polyester-based filaments, and in particular on the occurrence of unexpected shrinkage phenomena after post-processing heat treatment, are presented.

## 1. Introduction

Semicrystalline polylactide (PLA) is one of the best-known polymers with the ability to change its morphology. The PLA morphology can be altered either during processing or under load. PLA exhibits a glass transition temperature (*T_g_*) in the range of 55–70 °C and melting temperature (*T_m_*) in the range of 150–170 °C. The main disadvantage of PLA is its deformation at relatively low temperatures (above the *T_g_*) [1]. However, the high degree of crystallinity contributes to a significant improvement in PLA stability at higher temperatures [2]. PLA, which is the most widely used and thus, the best-known polymer among bio-based and/or (bio)degradable polymers, is also a suitable material for three-dimensional (3D) printing [3,4]. Polyhydroxyalkanoates (PHAs) are non-toxic bio-based, biodegrade, and biocompatible polyesters. The reported applications of PHAs in many different fields such as food packaging, drug delivery, tissue engineering, sutures, scaffolds, bone plates, agricultural foils, mulch films and 3D printing testify to the importance of this polymer. Recent advances in the use of PHA in 3D printing in the medical area include regenerative medicine, artificial tissues, and organs [4].

3D printing (additive manufacturing) is widely used in many sectors such as textile and consumer goods, agriculture and horticulture, electric and electronics, building and construction, automotive and transportation, aeronautic, medical engineering, packaging and food-service sectors for rapid prototyping and small-lot production. Due to high precision, high efficiency for a single product and convenient operation, 3D printing is becoming more popular not only in the medical, pharmaceutical and dental industries but also for personalized consumer products. 3D printing of biomedical devices and biocompatible structural components is more and more widely used and it is extremely important to precisely control the processing conditions, parameters and material deposition during printing in order to obtain structures that mimic natural ones. Not only element shape but also other factors such as contact time with the printer working platform during printing, printing orientation (build and raster), infill percent and pattern, cross-sectional area, layer height (resolution), and post-processing (methods and time) are important. Besides this, the relationship between the air pressure, feedrate, the nozzle distance to the printing platform and printing sharp angles of the expected structures, is also important. This means that the success of the 3D printing process also depends on an appropriate selection of process parameters [4,5,6,7,8].

Polymers during processing, tend not only to orient but they also shrink. In addition to shrinking due to normal thermal expansion (heat-induced contraction), sometimes the orientation may be unbalanced, causing distortions. Shrinkage begins at the molecular level when materials melt and cool and can be longitudinal or/and transverse. The primary shrinkage of semicrystalline polymers arises when the material phase goes from a melt (liquid) to solid, and a change in contraction rate occurs. The secondary shrinkage occurs primarily in the case of crystalline polymers since they undergo further structural transformation processes. Crystalline regions have a lower coefficient of expansion than amorphous regions. Crystallization can begin below the melting point and can last long after the element has cooled to room temperature (even for several days or weeks) [9]. Semicrystalline polymers shrink at a rate incomparably higher than the amorphous ones. In the relaxed state, the semicrystalline polymers are mostly structurally aligned, while the orientation of amorphous polymers is always completely random. The defects result from an unbalanced orientation with shrinkage. Orientation is rarely uniform across the cross-section of the element due to the various drawdowns, shear stress, and cooling rates throughout the element. In the case of highly-oriented materials, the unbalanced shrinkage can even be up to 50% of the length of the specimens, which causes serious distortions. The biggest problem is that this distortion may not appear for a long time if the element has been quickly quenched and is kept cool after processing. The process of secondary annealing, which allows complete relaxation of the polymer, can eliminate orientation in a processed element [10,11]. This undesirable phenomenon is also sometimes created during the 3D printing process, which involves reducing the volume of material under the impact of a rapid change in temperature. Shrinkage effect can be considered as a shape memory property because after permanent deformation under the impact of mechanical loads, the polymer can recover its original shape only by the action of temperature. In the case of semicrystalline polymers, the shape memory behavior is manifested by thermal retraction due to heating when the molecular structure has been oriented with a mechanical load. The shrinkage is anisotropic due to orientation. In particular, after the imposed strain, the shape remains immovable until the temperature is equal to or lower than the temperature of deformation. If the temperature is increased, a retraction occurs or shrinkage stress arises [12]. Too high temperatures can cause problems with shrinkage phenomena, such as sinking, warping, contraction and void formation. On the other hand, too low temperatures can promote flow marks, weld lines, poor surfaces, lamination, and short shots [13].

This scientific paper aims to provide detailed explanations of the original results obtained from a series of experiments that have investigated the behavior of a material with shrinkage phenomena. The purpose of this paper is to describe the defect that occurs during the degradation processes, how that impacts on the degradation mechanism and to provide explanations of these results. A variety of applications, especially clinical ones of (bio)degradable polymers, require a proper understanding of their properties and behavior during usage as well as during degradation. Throughout the preparation of various types of biomedical devices, and biocompatible structural components, certain conditions may occur, after they have been inserted into the patient’s body, which can lead to different types of damages and element failures. That is why it is so important to determine the impact of a specific processing method, i.e., processing parameters and conditions, on polymer properties and its degradation process expressed in molar mass and thermal properties changes [3,4].

## 2. Materials and Methods

### 2.1. Materials

The materials used in this study were two commercial 3D printing filaments: PLA filament (Orbi-Tech, Leichlingen, Germany) with a mass-average molar mass *M_w_* = 274,000 g/mol, molar-mass dispersity *M_w_*/*M_n_* = 2.4 (determined by gel permeation chromatography analysis) and *D*-lactide content equal to 5.2% and PLA/PHA filament (ColorFabb, Belfeld, The Netherlands) with 88 wt% of PLA (determined by thermogravimetric analysis) and *D*-lactide content equal to 5.0%. The PHA component in the PLA/PHA blend mainly contains 3-hydroxybutyrate units and a small number of 3-hydroxyvalerate units. However, it was not possible to differentiate/ascertain whether the PHA component is a poly(3-hydroxybutyrate-*co*-3-hydroxyvalerate) (PHBV) or a poly(3-hydroxybutyrate) (PHB)/PHBV blend. The detailed material characteristic was described in [4].

### 2.2. 3D Processing of Dumbbell-Shaped Specimens

3D printed PLA and PLA/PHA dumbbell-shaped specimens type 1BA (specimen geometries according ISO 527 standard [14]) were obtained using a fused deposition modelling printer (FLASHFORGE Dreamer dual extrusion 3D printer, FlashForge Corporation, Jinhua, China). Specimens with unexpected shrinkage phenomena after post-processing heat treatment were prepared according to [4,15] with two different processing build directions, one with a horizontal (flat, XY plane) processing build direction and with raster angle (45°/−45°) and a second with a vertical (upright, ZX plane) processing build direction and with raster angle (90°/0°). The detailed characteristics of the specimens without shrinkage phenomena, including molar masses and mechanical properties before and after degradation, was described in [4,15]. Specimens for investigating the impact of conditioning and various printer working platform and nozzle temperatures on shrinkage phenomena were printed in a horizontal processing build direction (flat, XY plane) with 7 perimeter layers and with oblique infill with raster angle (45°/−45°) in a wide section for better specimens strength. The constant printer settings used for printing the specimens were as follows: printing speed 40 mm/s, printing time 12 min nozzle diameter—400 µm, layer thickness—200 µm, infill pattern, and density—rectilinear, 100%.

### 2.3. Specimens Preparation and Conditioning Process (Annealing)

A series of dumbbell-shaped specimens with unexpected shrinkage phenomena after post-processing heat treatment were not conditioned prior to degradation and printed just before the degradation experiment. Therefore, the degradation test to repeat the shrinkage phenomena was carried out from specimens printed on the same day. Whole specimens and specimens cut in half with scalpel were taken for the degradation experiments. Due to the fact that some parts are printed with support material that will be cut off (especially in vertical printing), knowledge about the behavior of such materials may be important. Specimens for investigating the impact of conditioning and various printer working platform and nozzle temperatures on shrinkage phenomena were conditioned at four different temperature cycles of annealing: (i) 2 h at 110 °C; (ii) 1 h at 60 °C and 1 h at 110 °C; (iii) 1 h at 70 °C and 1 h at 110 °C or (iv) 2 weeks at 50 °C and 1 h at 110 °C.

### 2.4. Hydrolytic Degradation Experiments

(i) For the degradation experiment with unexpected shrinkage phenomena after post-processing heat treatment, the dumbbell-shaped specimens (whole and cut specimen) were incubated at 70 °C (±0.5) over a period of 70 days in demineralized water. Specimens were cut in half to check for differences in material degradation, as a mechanical break in its continuity could cause changes in degradation progress. The detailed study on the degradation experiment (without shrinkage phenomena) was described in [4,15]. (ii) For additional degradation experiments in which an attempt was made to find out the reasons for the shrinkage phenomena after post-processing heat treatment, the dumbbell-shaped specimens (whole and cut specimen) were incubated in the same conditions as before for a period of 7 days only, since the shrinkage had to occur in the initial stage of degradation after applying the elevated temperature. The specimens incubated were run in triplicate. After a predetermined degradation time, the specimens were separated from the degradation medium, washed with demineralized water and dried first on filter paper and then under vacuum at a temperature of 25 °C to a constant mass. The mass loss was performed with triplicate measurements and calculated as described by [16,17].

### 2.5. Characterization

#### 2.5.1. Scanning Electron Microscope (SEM)

SEM studies were performed using of a Quanta 250 FEG (FEI Company, Fremont, CA, USA) high resolution environmental scanning electron microscope operated at 5 kV acceleration voltages. The specimens were observed without coating under low vacuum (80 Pa) using a secondary electron detector (large field detector).

#### 2.5.2. Gel Permeation Chromatography Analysis (GPC)

The molar mass and molar-mass dispersity of the specimens were determined using gel permeation chromatography conducted in chloroform solution at 35 °C with an eluent flow rate of 1 mL/min with a set of two PL-gel 5 μm MIXED-C ultrahigh efficiency columns (Polymer Laboratories, Church Stretton, UK) with a mixed bed and a linear range of *M_w_* = 200–2,000,000 g/mol (for comparison of molar masses before and during the degradation process of specimens printed with various times and speeds of printing) as well as one Mixed E Styragel column with a mixed bed and a linear range up to *M_w_* = 25,000 g/mol (for comparison of molar masses of the specimens after 70 days of degradation). An isocratic pump (Spectra Physics 8800, Newport Corporation, Irvine, CA, USA) as the solvent delivery system and a differential refractive index detector stabilized to a temperature of 35 °C (Shodex SE 61, Showa Denko, Munich, Germany) was applied. 10 μL of 0.5% m/V sample solution was injected into the system. Polystyrene standards (Calibration Kit S-M-10, Polymer Laboratories) with narrow molar-mass dispersity were used to generate a universal calibration curve. The specimens were measured using OmniSEC 5.0 (Viscotek, Malvern, UK) software.

#### 2.5.3. Differential Scanning Calorimetry (DSC)

Thermal characteristics of the specimens were obtained using the TA-DSC Q2000 apparatus (TA Instruments, Newcastle, DE, USA). The instrument was calibrated with high purity indium. The first heating run concerned initial specimens in which the thermal history is suppressed. The second heating runs were performed for specimens previously melted and then cooled to −90 °C. DSC studies were carried out at a temperature from −90 to 200 °C at a heating run of 20 °C/min. All of the experiments were performed under a nitrogen atmosphere with the nitrogen flow run of 50 mL/min, using aluminum sample pans. The *T_m_* was taken as the peak temperature maximum of that melting endotherm, and the *T_g_* was taken as the midpoint of the heat capacity change of the specimen.

## 3. Results and Discussion

**Figure 1:**
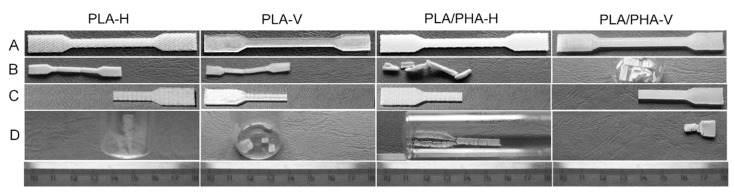
Macrographic images of the polylactide (PLA) and PLA/polyhydroxyalkanoate (PHA) dumbbell-shaped specimens obtained by 3D printing in horizontal (H) and vertical (V) directions before (A and C) and after 70 days of hydrolytic degradation test at 70 °C (B—the whole specimens after degradation, D—cut in half specimens after degradation).

During the standard examination of the hydrolytic degradation course of the 3D printed specimens, unexpected shrinkage phenomena after post-processing heat treatment (shrinkage and warping) were found for PLA and PLA/PHA dumbbell-shaped specimens, representing approximately 50% of the length of the specimens in one series of degradation (after a period of 70 days) (see Figure 1). Usually, PLA-based material disintegrates already at the initial stage of hydrolytic degradation (at 70 °C after 7 days) [4,15]. However, there was no significant disintegration in this case. In view of the fact that it is a quite significant defect, an attempt was made to find the reasons for this phenomenon and to examine its effect on the degradation process.

### 3.1. Failure Analysis and Characteristics of Dumbbell-Shaped Specimens with Unexpected Shrinkage Phenomena after Post-Processing Heat Treatment During Degradation Experiment

**Figure 2:**
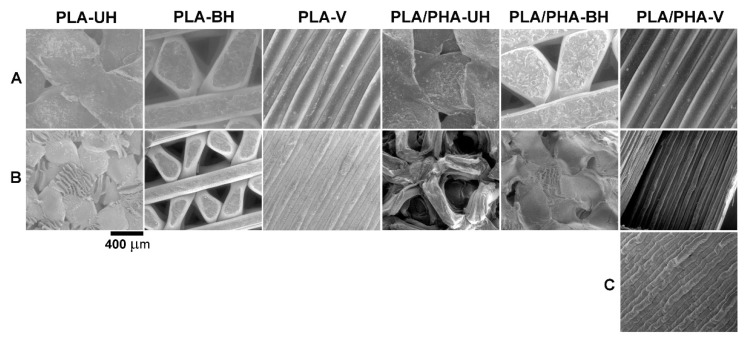
Selected SEM micrographs (200×) of the upper (UH) and underside (BH) layers of PLA and PLA/PHA dumbbell-shaped specimens obtained by 3D printing in horizontal (H) and vertical (V) directions before (**A**) and after 70 days of hydrolytic degradation test at 70 °C (**B**) the whole specimens after degradation, (**C**) cut in half specimens after degradation).

Dumbbell-shaped specimens with shrinkage phenomena were subjected to macro- and microscopic observation before and after degradation. After 70 days of hydrolytic degradation testing at 70 °C, PLA and PLA/PHA dumbbell-shaped specimens showed significant shrinkage phenomena, which represent approximately 50% of the length of the specimens (Figure 1 and Figure 2).

Based on organoleptic assessment due to the large disintegration of PLA dumbbell-shaped specimens obtained by 3D printing in horizontal and vertical directions (both cut in half), as well as PLA/PHA dumbbell-shaped specimens obtained by 3D printing in a vertical direction (whole specimen), it was difficult to ascertain whether shrinkage phenomena occurred (Figure 1). No shrinkage phenomena were observed for the PLA/PHA dumbbell-shaped specimen obtained by 3D printing in the horizontal direction (cut in half). The other specimens underwent contraction. In the case of PLA/PHA dumbbell-shaped specimens obtained by 3D printing in the horizontal direction (whole specimens), additional warping (twisting) of the specimens was observed, while additional stress caused by cutting the specimens in half resulted in quite different effects after degradation. The PLA dumbbell-shaped specimens (cut in half) were degraded to amorphous oligomers, which was then also confirmed by the DSC study, whereas the PLA/PHA dumbbell-shaped specimen obtained by 3D printing in vertical direction (cut in half) underwent an even larger shrinkage in the longitudinal direction.

Microscopic observation is a powerful tool to detect 3D printing defects. SEM images clearly show that the width of the filament decreased in the range from 41% to 66% (see Table 1) according to the following order: PLA-H > PLA/PHA-H > PLA-V > PLA/PHA-V for whole specimens and PLA/PHA-V > PLA/PHA-H for the cut in half specimens (considering the average of both layers for horizontal printing direction). It should also be noted that in the case of the PLA/PHA dumbbell-shaped specimen obtained by 3D printing in the vertical direction (whole specimen), and especially PLA/PHA dumbbell-shaped specimen obtained by 3D printing in the horizontal direction (cut in half), loss of filament width could only be caused by the degradation process and dissolution of degradation products as evidenced by the larger spaces between the filament in the specimens (Figure 1 and Figure 2). Two phenomena were observed: shrinkage effect and mass loss due to degradation and dissolution of degradation products.

Interestingly, for whole specimens, the largest contraction was observed in the case of PLA dumbbell-shaped specimens obtained by 3D printing in the vertical direction (51% of the overall length, Figure 1). For PLA dumbbell-shaped specimens obtained by 3D printing in the horizontal direction, the contraction was 40%. Shrinkage is a result of thermal and mechanical conditions that act on the final polymer material during processing. The shrinkage of extruded semicrystalline parts of thermoplastic polymers is influenced by the volumetric shrinkage, flow-induced residual stresses and orientation, flow-induced crystallization and heat transfer. The morphology of the material, especially in the case of heterophasic blends, plays a significant role in the shrinkage. Mixing of different polymers modifies the phase morphology, also by nucleation, which to some extent affects the shrinking effect. The shrinkage is related to the molar mass of the dispersed phase and the viscosity ratio between the components of the polymer matrix, respectively [18,19]. Nucleation usually results in an increase in the crystallization rate of polymers. This reduces the size of the crystals and causes an increase in their number and better dispersion [9]. In our case, the smaller shrinkage of the PLA/PHA specimens could have been affected by the nucleation effect due to the presence of the PHA component of the blend (acting as a nucleation agent) [4]. In the case of a blend, the warping effect of the filament itself was also observed (Figure 2). This did not affect the warping of the PLA/PHA dumbbell-shaped specimen obtained by 3D printing in the vertical direction (cut in half), but it affected the warping of the PLA/PHA dumbbell-shaped specimen obtained by 3D printing in the horizontal direction (whole specimen) probably due to the different degree of shrinkage of the individual components of the blend in the case of the absence of warping and individual filaments due to the printing geometry in the case of a horizontal direction.

**Figure 3:**
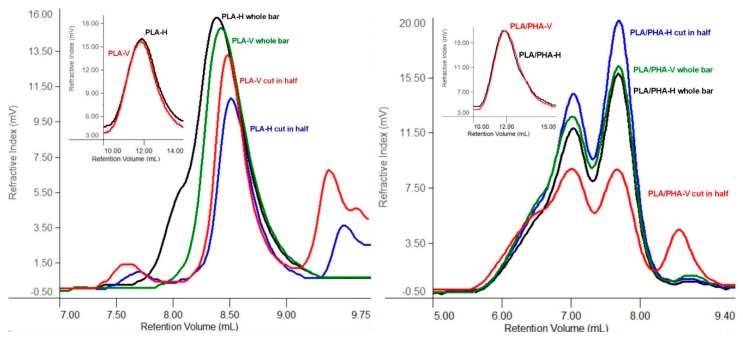
Overlay of selected gel permeation chromatography (GPC) chromatograms of PLA and PLA/PHA dumbbell-shaped specimens obtained by 3D printing in horizontal (H) and vertical (V) directions after 70 days of degradation at 70 °C (column with a linear range up to *M_w_* = 25,000 g/mol). A small picture shows the chromatogram of the material before degradation (two columns with a linear range of *M_w_* = 200–2,000,000 g/mol).

To assess the impact of shrinkage phenomena of the tested specimens on degradation after 70 days at 70 °C, GPC analysis was performed on columns for low-molar-mass polymers (linear range up to *M_w_* = 25,000 g/mol) (Figure 3). Molar masses of the specimens before degradation performed on columns for high-molar-mass polymers (linear range of *M_w_* = 200–2,000,000 g/mol) [15].

There were no significant differences in molar mass values between horizontal and vertical directions within each polymer, PLA as well as PLA/PHA blend. However, comparing PLA and PLA/PHA, a more significant shift towards low molar masses was observed in the case of the PLA dumbbell-shaped specimens (Figure 3). The smaller molar mass loss of PLA/PHA specimens resulted from the different degree of shrinkage of individual components of the blend, causing the effect of warping the filament itself and reducing the space between the filament, which limited the water penetration into the polymer matrix, and the removal of degradation products of low molar mass. Additional stress caused by cutting the specimens in half resulted in an increase in molar-mass dispersity, suggesting that cutting of both PLA dumbbell-shaped specimens and PLA/PHA dumbbell-shaped specimens obtained by 3D printing in a vertical (V) direction led to a greater shrinkage compared to non-cut ones, which further limited water penetration.

In order to evaluate the changes in the thermal properties of the tested specimens before and after degradation (as a consequence of the thermal history during the processing by 3D printing), DSC analysis was conducted (Table 2).

**Figure 4:**
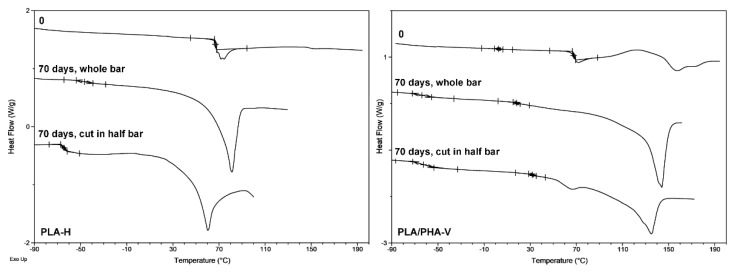
Selected differential scanning calorimetry (DSC) curves (first heating run) for the PLA dumbbell-shaped specimen obtained by 3D printing in horizontal (H) direction (**left**) and PLA/PHA dumbbell-shaped specimen obtained by 3D printing in vertical (V) direction (**right**) before (0) and after 70 days of hydrolytic degradation of whole and cut in half specimens.

In the first heating run for the PLA and PLA/PHA dumbbell-shaped specimens before degradation an endothermic phenomenon, the strong structural relaxation effect, due to enthalpic relaxation of amorphous glassy state from unstable chain conformations towards a more stable state, overlapped with PLA glass transition relaxation at approximately 63 ± 1 °C was observed (Table 2, Figure 4). The presence of an endothermic peak corresponding to the relaxation of ordered polymer chains, confirmed the orientation of the material during the printing process. The increase in the share of enthalpic relaxation indicated the internal ordering of the structure and the spontaneous pursuit of amorphous or semicrystalline material in order to achieve equilibrium, as well as higher thermodynamic stability at a given temperature (below the *T_g_*). Enthalpic relaxation is a parameter that characterizes the phenomenon of physical aging, during which the movements of some fragments/groups of molecules in the examined material vanish [20,21,22,23]. Then, on the DSC curve of PLA/PHA dumbbell-shaped specimens before degradation, a cold crystallization (*T_cc_*) with a lower temperature for horizontal processing build direction was observed (Table 2, Figure 4), confirming the earlier findings regarding the impact of the printing direction on the crystallization [4,24] and a melting endotherm with maximum temperature around 153 ± 1 °C for PLA and 172 ± 1 °C for PHA component of the blend. Since PHA similarly promotes crystallization as the nucleating agent [4,24], for PLA/PHA dumbbell-shaped specimens, enthalpy of cold crystallization (Δ*H_cc_*) was visible. 

During the second heating run, the PLA dumbbell-shaped specimens (previously melted and then cooled) did not exhibit a melting effect. In the case of PLA/PHA dumbbell-shaped specimens, the melting effect was lower. The first heating run provides information about the processing and the thermal and mechanical history of the specimen. In this run, heating the specimen to a temperature higher than the equilibrium of the melting point of a given polymer destroys all ordered regions so that the second heating run characterizes the polymer itself, without the effect of processing. The second heating run provides information on the structure of the polymer formed during cooling [25]. 

**Figure 5:**
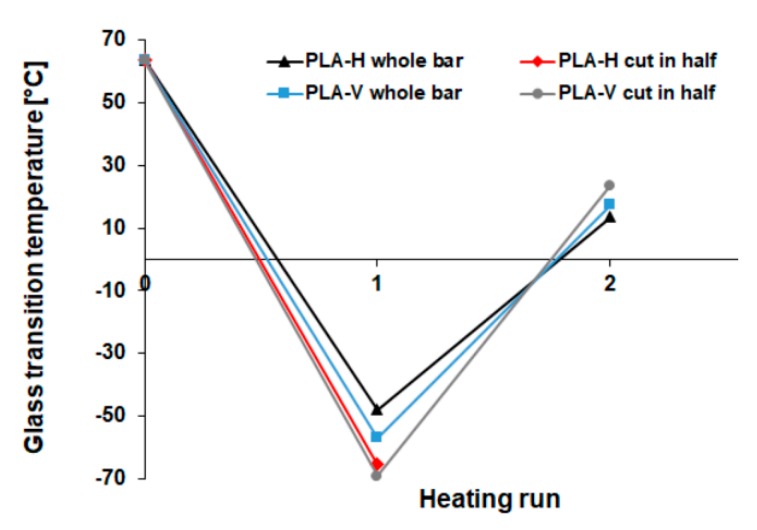
*T_g_* shift of PLA dumbbell-shaped specimens obtained by 3D printing in horizontal (H) and vertical (V) directions in subsequent heating runs.

PLA and PLA/PHA dumbbell-shaped specimens (whole and cut specimen), after 70 days of hydrolytic degradation test at 70 °C, showed that shrinkage phenomena differ in thermal properties. During the first heating run, melting was observed followed by an evaporation effect that suggested the evaporation of a part of the specimens, as confirmed by the recorded mass loss. In subsequent runs, further slow evaporation occurred, which resulted in a shift of *T_g_* of PLA component (also in the blend) towards higher values (Table 2, Figure 5). This indicated that the PLA and PLA/PHA dumbbell-shaped specimens have been degraded to amorphous oligomers and hydroxy acids [26], which evaporated during analysis causing an increase in *T_g_* of the PLA component.

In the first heating run, *T_g_* varied from −69.1 °C for PLA dumbbell-shaped specimens printed in the vertical direction (cut in half) with the largest molar mass loss to −48.0 °C for PLA dumbbell-shaped specimens printed in the horizontal direction (whole specimen) with the lowest molar mass loss. The biggest amount, up to 62% after the four runs (data not shown, 57% after three runs), evaporated for PLA dumbbell-shaped specimen printed in a horizontal direction (cut in half), which indicates a large amount of amorphous low-molar-mass degradation products, as also evidenced by the lowest *T_m_* = 60.4 °C. Usually, during hydrolytic degradation, the amorphous phase degrades faster, and *T_m_* increases, but in this case, a large amount of low-molar-mass amorphous degradation products were entrapped in the polymer matrix (which acted here as plasticizer) due to shrinkage and less water penetration into the matrix, resulting in a *T_m_* decrease. For other specimens, the mass loss was from 9% to 21% (after the third heating run, data not shown). The mass loss proceeded in the following order: cut in half PLA-H > PLA/cut in half PHA-V > cut in half PLA-V > PLA/cut in half PHA-H > PLA/PHA-H > PLA-V > PLA/PHA-V > PLA-H.

The broad melting profile characteristic of the presence of low-molar-mass degradation products was observed for all specimens except the PLA dumbbell-shaped specimen printed in a horizontal direction (whole specimen). For this specimen, the smallest mass loss of 9 % and the highest *T_g_* = −48.0 °C was also observed, which indicates that the polymer was more crystalline with a lesser amount of degradation products.

Cutting the specimen in half changed its orientation, which resulted in a change in behavior during degradation, causing a greater amount of low-molar-mass degradation products compared to non-cut specimens. This effect was the strongest for the PLA dumbbell-shaped specimen printed in the vertical direction (cut in half) where the lowest *T_g_* = −69.1 °C and the smallest melting enthalpy (Δ*H_m_* = 2.95 J/g) were observed.

In the second heating run, all PLA specimens exhibit low melting enthalpy, indicating a less ordered structure compared to PLA/PHA specimens.

### 3.2. Impact of External Conditions During Printing

The main factors affecting the shrinkage of the final item are the polymer structure and properties, processing parameters (times and speeds of printing, temperature, and cooling rate), the geometry of the element and its thickness. Differential cooling on the printer working platform can cause stress between different areas of the printed element and can also cause distortion upon removal from the printer [9]. The shrinkage phenomena can also be affected by various external factors, for example, the action of forces on the filament during printing, the temperature of the printer working platform, the partial nozzle closure (giving a too small amount of polymer), the pH of the degradation medium, the external temperature or the sun rays falling on the material. Therefore, the conditions during the 3D printing of specimens were analyzed.

The impact of partial nozzle closure (giving too small an amount of polymer) was eliminated by mass analyzing of the specimens that did not differ from the average. Over-heating of the specimens was simulated during printing by irradiating the specimens with an infrared heater to a temperature of 54 °C. The tests did not bring the expected result, on the contrary, the stress relaxation occurred, and there were no shrinkage phenomena after post-processing heat treatment.

Experiments were also carried out with a cooling fan, which is attached to the side of the print head and has a nozzle directed to the printing nozzle since a stream of cold air could cause rapid cooling of molten material. Then the maximum amount of stress could be accumulated in the stream of molten polymer, because there is no time for relaxation, as in the case of calm cooling. The shrinkage after 72 h of degradation in demineralized water at 70 °C (in the initial stage of degradation after applying elevated temperature) of cooled specimens during printing in the horizontal direction of PLA dumbbell-shaped specimens with and without construction lines (only infill) was 4 %, while without cooling 6% and 5% for specimens with construction lines and without, respectively (Table 3). Cooling did not have a major impact on the shrinkage that occurred, only to a small extent, mainly due to the crystallization of the specimens. The presence of construction lines also did not affect the shrinkage.

### 3.3. Impact of Printing Parameters: Times and Speeds of Printing

In order to analyze the effect of the action of forces on the filament during printing and the possibility of shrinkage during degradation experiments, dumbbell-shaped specimens were printed with various times and speeds of printing. The constant printer’s settings used to print specimens were as follows: working platform temperature—65 °C, nozzle diameter—400 µm, layer thickness—200 µm, infill pattern and density—rectilinear, 100%. Various printer settings used to print specimens and specimen’s dimensions are shown in Table 4.

The PLA dumbbell-shaped specimens printed with various times and speeds of printing were subjected to hydrolytic degradation for 7 days at 70 °C (in the initial stage of degradation after applying elevated temperature) to analyze the possibility of shrinkage similar to that observed during hydrolytic degradation with unexpected shrinkage phenomena after post-processing heat treatment. Unfortunately, during the experiments, no significant differences in the size of the specimens were noted. They all disintegrated, making it difficult to measure accurately. Disintegration indicates their fragility, while in the case of specimens with shrinkage phenomena, no such disintegration occurred even after 70 days of degradation (see Figure 1). Specimens also strongly disintegrated after 7 days of degradation in which there were no effects of shrinkage phenomena after post-processing heat treatment (see Figure 13 in publication [4] regarding the impact of contact time with the printer working platform, which leads to an increase in the crystalline phase during printing and affects the degradation process). Only the twisting of horizontally printed specimens, in particular PLA-50/5H specimen and small curvature (C-shaped) of vertically printed specimens, in particular PLA-50/15V and PLA-1/165V, were observed. Specimens printed with the longest time, 165 min for vertical direction and 210 min for the horizontal direction, have been disintegrated more than others, which indicates their greater fragility.

After 7 days of hydrolytic degradation in demineralized water with pH 5.5–5.6, no significant differences in molar mass of the PLA dumbbell-shaped specimens obtained by 3D printing at 1, 50 and 70 mm/s in horizontal and vertical directions were observed (data not shown). For all specimens, the molar mass loss was 97%–98%, which was in line with the degradation after 7 days for specimens without the effect of shrinkage phenomena in publication [4] (regarding the continuation of the study on the impact of printing parameters on degradation).

**Figure 6:**
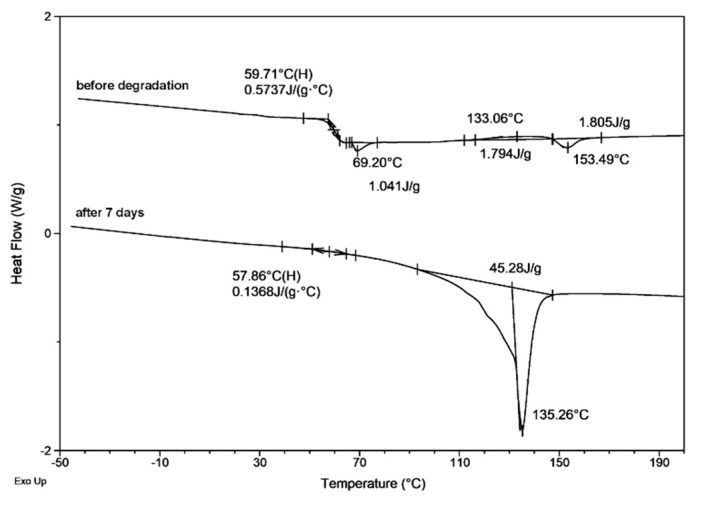
Selected DSC curves for the PLA dumbbell-shaped specimen PLA-1/165V-7, obtained by 3D printing at 1 mm/s and 165 min in vertical direction before and after 7 days of hydrolytic degradation.

No significant differences in thermal properties were found between specimens with shrinkage phenomena and those in which these phenomena did not occur. Furthermore, due to the lack of significant changes in thermal properties, if compared to the results of the previous publications [4,15], results were not presented. However, it is worth mentioning that for specimens printed at 50 mm/s for 5 min (PLA-50/5H-7) and 15 min (PLA-50/15V-7), no *T_g_* was observed. Additionally, for a specimen printed at 1 mm/s for 165 min (PLA-1/165V-7), the glass transition was small (Δ*c_p_* = 0.14 J/g°C), which indicates a strong crystallization of specimens after degradation. In the case of the specimens exhibiting the highest enthalpy of cold crystallization before degradation (PLA-1/165V-0 and PLA-1/210H-0, Δ*H_cc_* = 1.8 J/g and Δ*H_cc_* = 2.16 J/g, respectively), the greatest fragility was observed after degradation causing their greater disintegration (Figure 6).

### 3.4. Impact of Conditioning and 3D Printer Parameters: Working Platform and Nozzle Temperatures

The effect of conditioning, which should allow complete relaxation, on the specimens behavior during the degradation experiment and 3D printing conditions, in particular, various printer working platform and nozzle temperatures, were also examined to minimize the potential shrinkage that occurred during conditioning.

**Figure 7:**
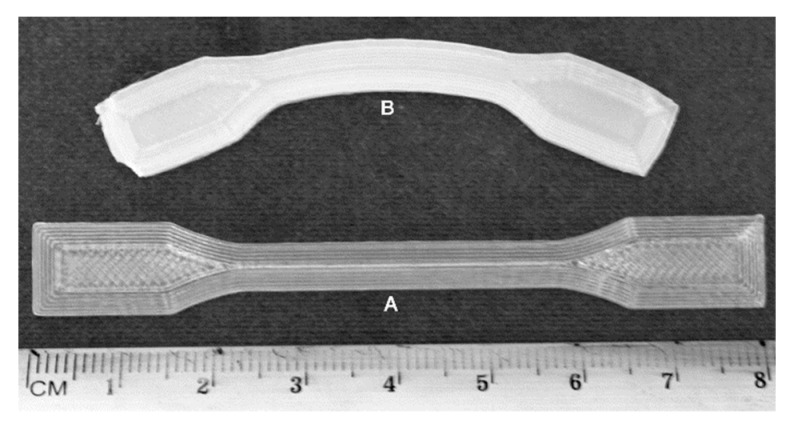
Macrographic image of PLA-60/200 specimen conditioned 2 h at 110 °C before (A) and after (B) placing at 110 °C.

The chosen printing pattern with 7 perimeter layers and oblique infill with raster angle (45°/−45°) in a wide section meant that, regardless of the conditioning method (2 h at 110 °C, 1 h at 60 °C and 1 h at 110 °C, 1 h at 70 °C and 1 h at 110 °C or 2 weeks at 50 °C and 1 h at 110 °C), all specimens printed with printer working platform and nozzle temperatures of 60 and 200 °C, respectively, have shrunk and deformed after being inserted to 110 °C of conditioning temperature (Figure 7).

To eliminate this effect, the 3D printing conditions were changed (Table 5), in particular, printing at different temperatures of the working platform and nozzle. As the printer working platform and nozzle temperatures rose from 70 and 220 °C to 120 and 240 °C, respectively, shrinkage and deformation decreased but were not completely eliminated.

Estimation of the polymer properties is strongly dependent on the processing history. When the material is cooled from above to below *T_g_*, the resulting glass is unstable, and its density gradually increases over time. This process toward thermodynamic equilibrium (structural relaxation) occurred faster at a temperature close to *T_g_* [27]. During melting, the polymers tend to have a random state, and the forces between the molecules weaken and move away from each other. Therefore, the crystalline structures relax, and the molecules adapt to the direction of flow. For crystalline polymers, after cooling below their crystallization temperature, the ordered structure is maintained in the direction of flow and the molecules begin to recrystallize, resulting in a significantly higher shrinkage rate. When these polymers cool down, the molecules form crystalline regions. This structure allows the material to fit together more tightly, making it denser and able to shrink more. In a state of equilibrium amorphous polymers have a random and entangled molecular orientation and require less realignment to achieve a relaxed state when cooled after melting. This is also justified by the small change in density for amorphous polymers from the melt to the solid state. This shrinkage effect may include all dimensional changes occurring during extrusion cooling or not. During processing, the polymers in the molten state are subjected to flow-induced stress orientation and mechanical stretching in the molten to a semisolid state during their cooling, which causes the molecular structure to become oriented proportional to the stress. The more polymer molecules that are oriented, the more shrinkage that will ultimately appear in that direction. The difference in transverse and machine direction shrinkage is a visible indicator of how much unbalanced orientation is in the processed element [10].

**Figure 8:**
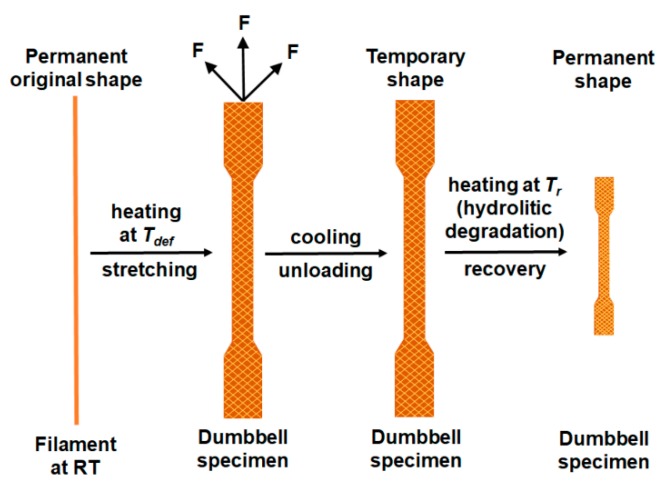
Shrinkage effect considered as thermal retraction; *T_def_*—deformation temperature, *T_r_*—temperature of retraction process.

After processing, shrinkage may begin longitudinally and/or transversely at the molecular level as the material first melts and then cools. The shrinkage may result from the high ordering of the polymer molecular structure associated with an increase in crystallinity during processing. Moreover, when printing at temperatures, not above the nozzle temperature but above *T_g_*, the forces acting on the filament cause tensile stress leading to the polymer orientation and crystallization. Increasing the temperature causes molecular motion, which gives typical rubber-elastic properties. A constant force applied to the polymer at temperatures above *T_g_* causes a viscoelastic deformation. During the 3D printing process, which involves reducing the volume of material under the impact of rapid temperature change after permanent deformation under the impact of mechanical loads (filament under force), the polymer can recover to its original shape due to temperature effects. In the case of semicrystalline polymers after the imposed strain, they remain immovable until the temperature is higher than the deformation temperature (thermal retraction due to heating). The specimens during degradation at 70 °C behaved such as purposely oriented material subjected to a thermal retraction test. The shrinkage phenomena can be caused by chemical (e.g., pH value), physical and thermal factors. It can be assumed that PLA as a thermoplastic polymer exhibits a shrinkage that can be considered as a shape memory property because the temperature is one of the main parameters (Figure 8).

## 4. Conclusions

It was originally found that the processing conditions, in particular, the contact time of the polymer material with the printer working platform, lead to an increase in the crystalline phase of the polymer during printing [4,15,24]. The current publication describes unexpected shrinkage phenomena after post-processing heat treatment observed during a standard degradation study of PLA and PLA/PHA dumbbell-shaped specimens in the degradation series after a period of 70 days, which represents about 50% of the length of the specimens. The shrinkage phenomena observed during the degradation experiment as a defect turned out to be significant and repeatable at a temperature of 110 °C. The unexpected shrinkage phenomena after post-processing heat treatment, occurred in both non-conditioned as well as in conditioned specimens, under specific environmental conditions (hydrolytic degradation at 70 °C and conditioning at 110 °C). Thermoplastic processing, included in the case described, heated the plastic filaments to a temperature above the melting point of the polymer, and then the material was cooled, which led to a shrinkage affected from three possible causes. First, when the material phase changed from melt to solid, a change in the contraction rate could occur. Second, PLA and PHA may crystallize below the melting point, and their volume changes upon crystallization. If the ambient temperature (temperature of degradation medium or conditioning environment) was higher than the glass transition temperature, this could cause unexpected warpage. Third, normal thermal contraction may also have occurred during cooling. The shrinkage phenomena have been also influenced by external factors such as the geometry of the element, the printer working platform and nozzle temperatures. It is also worth mentioning that the shape memory property manifested by thermal retraction could also play a significant role in PLA-based materials. Cutting the specimen in half disrupted its orientation, which resulted in a behavior change during degradation experiments. Furthermore, shrinkage phenomena caused an increased loss of molar mass during the degradation experiment, while on the other hand, cutting the dumbbell-shaped specimens in half led to an increase in molar-mass dispersity. The observation of unexpected shrinkage phenomena after post-processing heat treatment in the case of materials made of PLA or with its addition, which are of great importance in specialized applications, especially with potential for biomedical use, is a quite significant material defect.

## Figures and Tables

**Table 1:** Average filament width of the upper (UH) and underside (BH) layers of PLA and PLA/PHA dumbbell-shaped specimens obtained by 3D printing in horizontal (H) and vertical (V) directions before and after 70 days of hydrolytic degradation test at 70 °C.

Specimen	PLA-H		PLA-V	PLA/PHA-H		PLA/PHA-V
	Upper Layer	Underside Layer		Upper Layer	Underside Layer	
	Average Filament Width (μm) (Width Loss (%))					
Before degradation	664 ± 103	481 ± 117	213 ± 38	666 ± 116	488 ± 91	210 ± 33
After degradation, the whole specimen	394 ± 26 (41)	221 ± 45 (54)	96 ± 13 (55)	386 ± 74 (58)	196 ± 34 (40)	71 ± 12 (66)
After degradation, cut in half specimen	N/A	N/A	N/A	268 ± 54 (45)	161 ± 40 (76)	87 ± 18 (58)

N/A—not available.

**Table 2:** Thermal properties before and after 70 days of hydrolytic degradation of PLA and PLA/PHA dumbbell-shaped specimens obtained by 3D printing in horizontal (H) and vertical (V) directions (heating rate: 20 °C/min).

Specimen	Dumbbell-Shaped Specimens before Degradation				Dumbbell-Shaped Specimens after 70 Days of Degradation							
	PLA-H	PLA-V^a^	PLA/ PHA-H	PLA/ PHA-V^a^	PLA-H Whole Specimen	PLA-H Cut in Half Specimen	PLA-V Whole Specimen	PLA-V Cut in Half Specimen	PLA/PHA-H Whole Specimen	PLA/PHA-H Cut in Half Specimen	PLA/PHA-V Whole Specimen	PLA/PHA-V Cut in Half Specimen
First heating run												
*T_g_* (°C)	63.7	63.5	4.8/62.4	2.9/62.8	−48.0	−65.4/0.4	−56.9	−69.1/24.1	−62.1/−4.4	−63.4/2.5	−64.0/18.1	−61.6/31.5
Δ*c_p_* (J/g°C)	0.53	0.46	0.06/0.47	0.05/0.47	0.17	0.42/0.08	0.41	0.45/0.19	0.11/0.06	0.13/0.10	0.26/0.13	0.41/0.13
Enthalpic relaxation (°C)	74.5	72.5	68.7	72.0	–	–	–	–	–	–	–	–
*T_m_* (°C)	152.1	153.2	152.6/172.1	154.1/172.5	81.2/100.7	60.4	74.8/103.7	102.1	73.0/144.2	141.0	144.1	65.6/127.4/135.1
Δ*H_m_* (J/g)	0.51	1.29	18.10	13.32	52.6/0.05	52.41	63.69/3.01	2.95	72.82	86.8	80.06	4.29/53.11
*T_cc_* (°C)	–	–	111.7	124.9	–	–	–	–	–	–	–	–
Δ*H_cc_* (J/g)	–	–	−17.75	−13.11	–	–	–	–	–	–	–	–
Second heating run												
*T_g_* (°C)	62.4	61.9	2.0/59.5	3.0/60.8	13.6	N/A	17.4	23.5	−8.6	−4.6	−26.2	−21.1/27.8
Δ*c_p_* (J/g°C)	0.49	0.52	0.06/0.50	0.06/0.43	0.57		0.55	0.36	0.32	0.24	0.34	0.41/0.02
*T_m_* (°C)	–	–	152.6/174.5	154.2/171.7	95.2		114.8	116.0	135.7	135.9	120.2/135.5	66.6/102.0/127.8
Δ*H_m_* (J/g)	–	–	0.08/0.22	0.73	0.74		1.77	1.41	65.72	67.58	69.59	0.08/39.61
*T_cc_* (°C)	–	–	145.7	143.1	–		72.6	–	79.3	–	–	–
Δ*H_cc_* (J/g)	–	–	−0.32	−0.65	–		−1.54	–	−12.10	–	–	–

*T_g_*—glass transition temperature, Δ*c_p_*—the increment of heat capacity at the glass transition, *T_m_*—melting temperature, Δ*H_m_*—melting enthalpy, *T_cc_*—maximum of the exothermic peak of the cold crystallization temperature, Δ*H_cc_*—cold crystallization enthalpy, ^a^—average values from both sides of the dumbbell, N/A—not available.

**Table 3:** The results of the experiment with the cooling fan.

Parameters	Overall Length (mm)	Overall Width (mm)	Thickness (mm)	Overall Length (mm)	Overall Width (mm)	Thickness (mm)
	Before			After 72 h		
Cooling 2/2/2^a^	87.9 ± 0.3	10.3 ± 0.2	2.39 ± 0.1	84.0 ± 0.8	9.94 ± 0.3	2.35 ± 0.2
Without cooling 2/2/2^a^	88.0 ± 0.4	10.0 ± 0.2	2.27 ± 0.06	82.3 ± 0.7	9.71 ± 0.2	2.38 ± 0.1
Cooling 0/0/0^b^	87.7 ± 0.3	10.0 ± 0.2	2.38 ± 0.1	83.1 ± 0.6	9.62 ± 0.1	2.27 ± 0.1
Without cooling 0/0/0^b^	87.4 ± 0.2	9.7 ± 0.1	2.19 ± 0.1	82.6 ± 0.7	9.47 ± 0.2	2.24 ± 0.07

^a^—top solid layer/bottom solid layer/border (2/2/2), ^b^—only infill (0/0/0).

**Table 4:** Printer settings for preparation PLA and PLA/PHA dumbbell-shaped specimens obtained by 3D printing in horizontal (H) and vertical (V) directions and average specimen’s dimensions.

Specimen	Printing Speed (mm/s)	Printing Time (min)	Nozzle Temperature (°C)	Overall Length (mm)	Thickness (mm)	Mass (g)
Dumbbell-shaped specimens with shrinkage phenomena during degradation experiment						
PLA-H and PLA/PHA-H	50	15	200	88 ± 0.4	1.80 ± 0.01	0.99 ± 0.01
PLA-V and PLA/PHA-V	70	40	195	90 ± 0.4	2.07 ± 0.01	1.36 ± 0.03
Dumbbell-shaped specimens printed with various times and speeds of printing						
PLA-1/210H	1	210	200	87 ± 0.3	1.52 ± 0.01	1.21 ± 0.04
PLA-1/165V	1	165	195	91 ± 0.4	1.00 ± 0.06	0.90 ± 0.01
PLA-50/5H	50	5	200	87 ± 0.3	1.59 ± 0.07	1.26 ± 0.04
PLA-50/15V	50	15	195	91 ± 0.4	1.71 ± 0.20	1.44 ± 0.03
PLA-50/14H	50	14	200	88 ± 0.3	1.69 ± 0.01	1.31 ± 0.04
PLA-70/36V	70	36	195	93 ± 0.5	2.06 ± 0.03	1.66 ± 0.03

**Table 5:** Selected working platform and nozzle temperatures.

Specimen	Working Platform Temperature (°C)	Nozzle Temperature (°C)
PLA-60/200	60	200
PLA-70/220	70	220
PLA-80/210	80	210
PLA-90/220	90	220
PLA-100/230	100	230
PLA-120/240	120	240
